# Planthopper-Secreted Salivary Calmodulin Acts as an Effector for Defense Responses in Rice

**DOI:** 10.3389/fpls.2022.841378

**Published:** 2022-02-28

**Authors:** Jianmei Fu, Yu Shi, Lihua Wang, Tian Tian, Jing Li, Lei Gong, Zhouting Zheng, Maofeng Jing, Jichao Fang, Rui Ji

**Affiliations:** ^1^Institute of Plant Protection, Jiangsu Academy of Agricultural Sciences, Jiangsu Key Laboratory for Food Quality and Safety-State Key Laboratory Cultivation Base of Ministry of Science and Technology, Nanjing, China; ^2^College of Plant Protection, Nanjing Agricultural University, Nanjing, China; ^3^Jiangsu Collaborative Innovation Center of Regional Modern Agriculture and Environmental Protection, Huaiyin Normal University, Huai’an, China

**Keywords:** calmodulin (CaM), effector, planthopper, plant-insect interaction, rice defense response

## Abstract

The brown planthopper (*Nilaparvata lugens*, BPH) and small brown planthopper (*Laodelphax striatellus*, SBPH) are major pests of rice (*Oryza sativa*) in Asia. These piercing-sucking insects secrete saliva into the host during feeding. Nevertheless, it is largely unknown how planthoppers use salivary effectors to enable continuous feeding on rice. Here, we screened their salivary proteomes and selected eight salivary proteins conserved between SBPH and BPH as candidate effectors. Silencing *calmodulin* (*CaM*) impeded BPH and SBPH from penetrating the phloem. Hence, their food intake, survival, and fecundity on rice plants were reduced. By contrast, *CaM* silencing had a small effect on the survival rate of BPH and SBPH raised on artificial diet. The CaM amino acid sequences were the same for both BPH and SBPH. CaM was highly expressed in their salivary glands and secreted into the rice plants during feeding. Bacterially expressed recombinant CaM protein exhibited calcium-binding activity. *In planta* expression disclosed that CaM was localized to the plant cytoplasms and nuclei and suppressed plant defenses such as hydrogen peroxide (H_2_O_2_) accumulation and callose deposition. *CaM-*silenced BPH and SBPH nymphs elicited relatively high levels of H_2_O_2_ and callose accumulation in rice plants. The foregoing results reveal that CaM is an effector as it enables the planthopper to reach the phloem by suppressing callose deposition and H_2_O_2_ accumulation in rice.

## Introduction

Plants and herbivorous insects have been engaged in a long-term arms race. Plants have developed defensive mechanisms, but insects have evolved various strategies to counteract them. Plant cells can perceive herbivore-associated molecular patterns, rapidly elevate their cytosolic calcium (Ca^2+^) concentrations, a universal secondary messenger in eukaryotic organisms, and activate various defense responses such as phytohormone and hydrogen peroxide (H_2_O_2_) biosynthesis and sieve tube plugging (Arimura and Maffei, 2010; Ranf et al., 2011). While feeding, however, insects inject several salivary effectors into host plant cells and interfere with these host plant defenses (Ji et al., 2017, 2021; Ye et al., 2017; Huang et al., 2019; Tian et al., 2021). The identification of insect salivary proteins may help elucidate the mechanisms by which insects modulate host plant defenses. Recent research has generated abundant information regarding insect effectors and elicitors, especially those produced by piercing-sucking insects. These substances may positively (Ji et al., 2017, 2021; Ye et al., 2017; Huang et al., 2019; Wang et al., 2019; Xu et al., 2019; Tian et al., 2021) or negatively (Bos et al., 2010; Chaudhary et al., 2014; Rao et al., 2019; Guo et al., 2020) affect insect feeding performance on plant hosts. Such interactions resemble those occurring between microbial pathogens and their host plants.

The accumulation of H_2_O_2_ and cell wall polymer callose are common and effective plant defenses against sap-sucking insects including rice planthoppers. H_2_O_2_ is associated with lignin formation in cell wall, and inhibits insects feeding (Rasool et al., 2017). The deposition of the β-1,3 glucan polymer callose on the sieve plates causes sieve element sealing, and prevents insects from sucking phloem sap (Hao et al., 2008). When certain salivary effectors are secreted into plants, they attenuate these host defense mechanisms (Zhou et al., 2009; Ye et al., 2017; Huang et al., 2019; Su et al., 2019; Guo et al., 2020; Wang et al., 2020). For example, the mixture of aphid watery salivary proteins prevents Ca^2+^-dependent sieve elements plugging (Will et al., 2007). Salivary Mp55 from the aphid and LsDNase II from the planthopper suppresses insect-elicited callose deposition and H_2_O_2_ accumulation (Elzinga et al., 2014; Huang et al., 2019). The planthopper salivary effectors, such as vitellogenin and Ca^2+^-binding proteins, reported in our previous studies (Ye et al., 2017; Ji et al., 2021; Tian et al., 2021) suppress insect-elicited H_2_O_2_ accumulation by interacting with a plant immunity regulator and binding free Ca^2+^, respectively. Glutathione peroxidase from the mirid bug and ferritin from the whitefly suppress the plant H_2_O_2_ signaling pathway (Su et al., 2019; Dong et al., 2020).

The brown (*Nilaparvata lugens*, BPH) and small brown (*Laodelphax striatellus*, SBPH) planthoppers are sap-sucking insects that directly feed on rice (*Oryza sativa*) phloem and indirectly transmit viruses to the host. Hence, planthoppers cause major annual rice grain yield losses (Otuka et al., 2010). Saliva proteomic analyses identified certain salivary proteins from the two foregoing planthopper species (Huang et al., 2016, 2018; Fu et al., 2021). A few successive defense/counter-defense mechanisms may be observed in BPH, SBPH, and rice, and they illustrate the co-evolutionary processes underlying rice-planthopper interactions (Ji et al., 2017, 2021; Ye et al., 2017; Shangguan et al., 2018; Huang et al., 2019; Huang J. et al., 2020; Fu et al., 2021; Tian et al., 2021). In some cases, Ca^2+^ is required for the accumulation of H_2_O_2_ and callose in plant (Hayashi et al., 1987; Zuppini et al., 2004). Salivary elicitors disulfide isomerase (LsPDI1) and mucin-like protein from planthoppers induce plant immune responses including cell death, H_2_O_2_, and callose accumulation that depend on the Ca^2+^ signaling pathway (Shangguan et al., 2018; Rao et al., 2019; Huang J. et al., 2020; Fu et al., 2021). Conversely, two Ca^2+^-binding proteins in the saliva of planthoppers attenuate Ca^2+^-triggered H_2_O_2_ biosynthesis by binding free Ca^2+^ in host rice (Ye et al., 2017; Tian et al., 2021). In aphids, injection of salivary Ca^2+^-binding proteins into hosts might be a universal mechanism for preventing sieve plate occlusion (Will et al., 2009). Nevertheless, certain salivary Ca^2+^-binding protein in phloem feeders including planthoppers preventing sieve tube occlusion, including callose deposition, remain to be established.

Interestingly, analysis of the saliva proteomes (Huang et al., 2018; Fu et al., 2021) and previously reported effectors from BPH and SBPH revealed that most of the effectors are, in fact, conserved salivary proteins. Here, we aimed principally at the eight salivary proteins conserved between SBPH and BPH to screen for candidate planthopper effectors. The RNA interference (RNAi) approach disclosed that only calmodulin (CaM) contributed to SBPH and BPH feeding. CaM has been detected in the saliva proteomes of various piercing-sucking herbivorous arthropods such as aphids, whiteflies, and spider mites but not yet in those of planthoppers (Huang et al., 2018; Huang H. J. et al., 2020; Fu et al., 2021). Therefore, CaM might play an important role in herbivore-plant interactions. Subsequent analyses demonstrated that CaM has an effector function and may facilitate planthoppers continuous feeding by inhibiting callose deposition and H_2_O_2_ accumulation in host rice plants. Thus CaM is a good candidate for RNAi-based planthoppers management.

## Materials and Methods

### Plant Growth and Insect Rearing

Pre-germinated Xiushui 11 rice seeds were germinated in plastic bottles in a greenhouse at 28 ± 2°C under a 14 h light/10 h dark photoperiod. Each 7-day-old seedling was then transferred to a 750 mL plastic pot containing potting medium. After 25 days, the rice plants were ready for use in the subsequent experiments. *Nicotiana benthamiana* was kept in a climate chamber at 23 ± 1°C under a 16 h light/8 h dark photoperiod. After 4–5 weeks (five-leaf stage), the plants were used in the subsequent *Agrobacterium tumefaciens*-mediated transient transformation experiments. The original SBPH colonies were obtained from rice fields in Nanjing, China and maintained on rice seedlings in a climate chamber at 25 ± 1°C under a 14 h light/10 h dark photoperiod. To minimize interference from egg deposition, fourth-instar nymphs were injected and metamorphosed into fifth-instar nymphs 2 days later, and the latter were used for rice defense response analyses. Injected third-instar nymphs were used for the survival analysis. Newly emerged brachypterous female adults within 24 h were used for honeydew excretion measurement and electrical penetration graph (EPG) analysis, owing to the highest feeding capacity at this stage.

### RNA Preparation and Quantitative Polymerase Chain Reaction

Total RNA was extracted from whole bodies of the first- to fifth-instar nymphs and newly emerged brachypterous male and female adults. It was also extracted from salivary glands, guts, ovaries, fat bodies, and integuments dissected from brachypterous female adults. For each biological replicate, 50 nymphs, 20 adults, and various tissues from 200 brachypterous female adults were collected. Each experiment was repeated in triplicate. Total RNA was isolated with the SV Total RNA Isolation System (Promega, Madison, WI, United States) according to the manufacturer’s instructions. After DNase treatment and quantification, 1 μg total RNA sample was reverse-transcribed with a PrimeScript RT-PCR Kit (TaKaRa Bio Inc., Dalian, China). The quantitative polymerase chain reaction (qPCR) reactions were performed with a TB Green™ Premix Ex Taq™ Kit (TaKaRa Bio Inc.) according to the manufacturer’s protocol and run in a LightCycler^®^ 480 II Real Time System (Roche Diagnostics, Basel, Switzerland). Elongation factor 2α (*ef2*) served as the internal normalization controls. The qPCR primers are listed in Supplementary Table 1. The gene expression levels were calculated by normalizing the target gene mRNA level to *ef2* mRNA abundance *via* the 2^–Δ*Ct*^ method (Chyla et al., 2019; Tian et al., 2021).

### Gene Cloning and Vector Construction

*LsCaM* and *NlCaM* (GenBank no. RZF46934.1 and AXY40108.1) were obtained by reverse transcription polymerase chain reaction (RT-PCR) using total RNA from SBPH and BPH. They were cloned into the pMD19-T vector (TaKaRa Bio Inc., Kusatsu, Japan), and sequenced. An In-Fusion HD Cloning Kit (TaKaRa Bio Inc.) was used to insert *LsCaM* into the pBINPLUS-green fluorescent protein (GFP) vector (subcellular localization and expression) *via* a single *Bam*HI digestion site. *LsPDI1* was cloned *via* homologous recombination into the pBINPLUS-mCherry vector (expression) *via* a single *Bam*HI digestion site using the aforementioned kit. Primers are listed in Supplementary Table 1.

### Double Stranded RNA Synthesis and Injection in Planthoopers

Primers containing the T7 promoter (Supplementary Table 1) were designed to clone a specific fragment (∼300–500 bp) in each target gene. The double stranded RNA (dsRNA) was synthesized and microinjected as previously described (Liu et al., 2010; Ji et al., 2017). To determine RNAi efficiency, target gene expression was measured at 2, 4, and 6 days after dsRNA injection. qPCR was repeated three times with six replicates per time, there were 20 insects per replicate.

### Planthopper Bioassays

Planthopper feeding behavior was recorded on a direct-current EPG system (Wageningen Agricultural University). The method used was previously described (Cao et al., 2013; Ji et al., 2017). The feeding behavior of newly emerged adult females 2 days after dsRNA injection was monitored for 6 h. There were 15 replicates per treatment. The recorded signals were analyzed with PROBE software (Wageningen Agricultural University). The durations of each sequential waveform event were measured for each insect. The average waveform duration per insect per waveform was calculated for each treatment.

Honeydew excretion reflects feeding activity in sap-sucking insects. To assess the effect of *CaM* knockdown on planthopper honeydew excretion, one rice stem was covered with an inverted transparent plastic cup placed on a filter paper. Three days after injection, a single newly emerged brachypterous female adult was transferred to the cup and left to feed for 2 days. The filter papers were then soaked in 0.1% (w/v) ninhydrin in acetone and oven-dried at 65°C for 30 min. The honeydew stains appeared as violet or purple spots because of their amino acid content (Lei et al., 2014; Ji et al., 2021). The areas of the ninhydrin-positive deposits were measured with ImageJ v. 1.8.0 (National Institutes of Health, Bethesda, MD, United States). There were 15 replicates per treatment.

The injected third-instar nymphs were left to recover on rice seedlings for 1 day. Healthy nymphs were randomly selected for the subsequent survival bioassay on rice plants or artificial diet. One rice stem per pot was confined within a glass cylinder (diameter, 2 cm; height, 8 cm) containing 20 third-instar nymphs. In the artificial diet experiment, 20 third-instar nymphs were introduced into individual feeding chambers (diameter, 2 cm; long, 9 cm) as described previously (Ji et al., 2017). The surviving nymphs were counted daily. The survival rate of each treatment and the corrected survival rates of nymphs with injected *dsLsCaM*, using nymphs with injected *dsGFP* as controls, on rice or artificial diet were calculated. The experiment was repeated five times.

The number of eggs laid per female adult was determined using a previously reported method (Ji et al., 2017). A single newly emerged dsRNA-treated female adult and two newly emerged untreated male adults were released into the same glass cylinder confining a single rice stem. The insects were allowed to feed for 8 days. The number of eggs laid by a female adult on each dissecting rice stem was counted under a microscope (Olympus SZ51, Olympus Corp., Tokyo, Japan). There were 15 replicates per treatment.

### Calmodulin Sequence Analysis

Signal peptides and transmembrane and EF-hand domains were predicted with SignalP v. 5.0,^1^ TMHMM v. 2.0,^2^ and PROSITE,^3^ respectively.

### Ca^2+^-Binding Assays

The complete open reading frame (CORF) of *LsCaM* was cloned into a pET-28a vector (Novagen, Madison, WI, United States). The LsCaM:pET-28a construct was transformed into *Escherichia coli* BL21 (DE3). The recombinant protein products were purified with Ni-NTA columns (Qiagen, Venlo, Netherlands) according to the manufacturer’s instructions. They were then concentrated with a YM-3 Microcon centrifugal filter device (EMD Millipore, Billerica, MA, United States).

The Ca^2+^-binding property of the recombinant CaM was determined by gel mobility shift assay as previously described (Anisuzzaman et al., 2010; Hattori et al., 2012; Ye et al., 2017; Tian et al., 2021). Briefly, 0.75 μg CaM was mixed with equal volumes of 0.5 mM CaCl_2_, 0.15 mM CaCl_2_, 0.05 mM CaCl_2_, 0.015 mM CaCl_2_, or 0.5 mM ethylenediaminetetraacetic acid (EDTA). Each mixture was incubated at 25°C for 30 min, combined with Laemmli sample buffer, and subjected to SDS-PAGE with no heating.

### Western Blotting

Proteins were extracted from the salivary glands of 100 fifth-instar SBPH or BPH nymphs, and from the leaf sheaths of rice steams confined within ventilated glass cylinders containing 200 fifth-instar nymphs that had been removed after 2 days. Leaf sheaths from rice stems in empty glass cylinders served as negative controls. The outer three leaf sheaths of each rice stem were harvested and pulverized in liquid nitrogen. Then 2 mL NP40 buffer (Beyotime Institute of Biotechnology, Shanghai, China) was added and the suspension was vortexed at 4°C for 20 min. Each sample was centrifuged at 15,200 × *g* and 4°C for 5 min. Each supernatant was collected and concentrated to 200 μL with a YM-3 Microcon centrifugal filter device (EMD Millipore, Billerica, MA, United States). The samples were subjected to SDS-PAGE on a 12% (w/v) gradient gel (Bio-Rad Laboratories, Hercules, CA, United States) and transferred onto a polyvinylidene difluoride (PVDF) membrane. Western blotting was performed using primary antibody (1:2,000 dilution) and visualized with the VersaDoc imaging system (Bio-Rad Laboratories) as previously described (Ji et al., 2017; Tian et al., 2021).

### Transient Calmodulin Expression in *Nicotiana benthamiana* and Rice Protoplast

Empty pBINPLUS-GFP or pBINPLUS-mCherry vector or their recombinant plasmids harboring CaM or LsPDI1 were transformed by electroporation into *A. tumefaciens* GV3101. The *A. tumefaciens* was re-suspended in an infiltration buffer (10 mM MgCl_2_, 500 mM MES, and 100 mM acetosyringone). At a final *OD*_600_ = 0.4, the *A. tumefaciens* was infiltrated into *N. benthamiana* leaves with a needleless syringe as previously described (Dong et al., 2020; Ji et al., 2021). The CaM-GFP was expressed for 24 h, then LsPDI1-mCherry was infiltrated into the same region. GFP (488 nm) and mCherry (561 nm) fluorescence signals were observed under a Zeiss LSM750 confocal laser-scanning microscope (CLSM; Carl Zeiss AG, Oberkochen, Germany). At 24 h post-LsPDI1 infiltration, the infiltrated *N. benthamiana* leaves were collected, and vacuum-infiltrated with 1 mg mL^–1^ 3,3′-diaminobenzidine (DAB, pH 7.2), and incubated overnight in the dark, and decolorized with trichloroacetaldehyde. DAB staining was performed on 20 leaves.

Rice protoplasts were isolated and transfected as previously described (Zhang et al., 2011; Ji et al., 2021). Briefly, 10-day-old rice seedlings were cut into 0.5 mm segments, transferred to 0.6 M mannitol, and incubated in the dark for 30 min. The plasmolyzed tissues were transferred to a mixture comprising 1.5% (w/v) Cellulase R-10 (Yakult Honsha, Tokyo, Japan), 0.75% (w/v) Macerozyme R-10 (Yakult Honsha), 0.5 M mannitol, 10 mM MES (pH 5.7), 0.1% (v/v) bovine serum albumin (BSA), 10 mM CaCl_2_, and 5 mM β-mercaptoethanol for cell wall degradation. Polyethylene glycol-mediated transfection was used to transform the recombinant vector with 2 × 10^6^ rice protoplasts. They were incubated in the dark for 16 h, and their green fluorescence was examined under a Zeiss LSM750 CLSM (Carl Zeiss AG).

### Callose Staining

Infiltrated tobacco leaf disks and planthopper-infested rice leaf areas were stained with aniline blue to visualize callose deposition as previously described (Bing et al., 2012; Fu et al., 2021). Briefly, treated samples were subjected to 0.05% (w/v) aniline blue staining for 2 h followed by 2.5 g/mL trichloroacetic acid decolorization. The treated samples were observed and photographed under a Zeiss LSM750 CLSM (Carl Zeiss AG) fitted with a UV filter. Fluorescence was quantified with ImageJ v. 1.8.0 (NIH). Each treatment was scored for 20 randomly selected microscopic fields. Each experiment was repeated six times.

### H_2_O_2_ Analysis

Samples were prepared for H_2_O_2_ analysis as previously described (Lou and Baldwin, 2006; Ji et al., 2017, 2021; Ye et al., 2017). Each single rice stem was confined in a glass cylinder containing 30 fifth-instar nymphs that had been injected with the double stranded RNA of *LsCAM* (*dsLsCAM*) or *GFP* (*dsGFP*) 2 days earlier. The outer two leaf sheaths of each stem were harvested at various time points after the treatment. Infiltrated *N. benthamiana* leaf disks and rice leaf sheath samples were collected for H_2_O_2_ measurement. H_2_O_2_ concentration was determined with an Amplex-Red Hydrogen Peroxide/Peroxidase Assay Kit (Invitrogen, Carlsbad, CA, United States) (Lou and Baldwin, 2006). Each experiment was repeated six times.

### Data Analysis

Differences in insect bioassay, genes expression, and H_2_O_2_ content among treatments were analyzed by one-way ANOVA followed by Duncan’s multiple range tests for multiple treatment comparisons or by Student’s *t*-tests for pairwise treatment comparisons. Data analyses were performed using Statistica v. 6 (SAS Institute Inc., Cary, NC, United States).

## Results

### Effect of Silencing Salivary Protein-Encoding Genes on Small Brown Planthopper Survival

**FIGURE 1:**
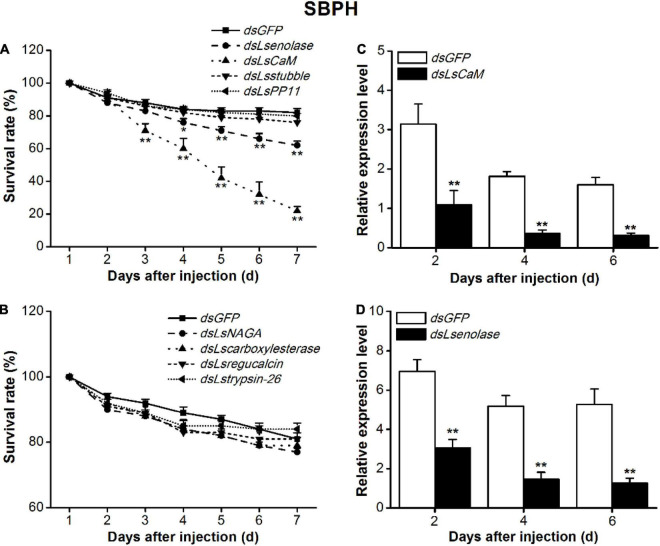
Effects of silencing salivary protein-encoding genes on survival rates of SBPH on rice. **(A,B)** Mean survival rates + SE (*n* = 5) of SBPH nymphs injected with dsRNA of indicated genes or *GFP* (*dsGFP*) at third-instar nymph stage. CaM, calmodulin; PP11, placental protein 11; NAGA, α-*N*-acetylgalactosaminidase. **(C,D)** Mean transcript levels + SE (*n* = 3) of indicated target genes in SBPH injected with dsRNA of *LsCaM* (*dsLsCaM*, **C**), *Lsenolase* (*dsLsenolase*, **D**), or *dsGFP*. Asterisks indicate significant reduction in survival rates **(A,B)** and gene expression levels **(C,D)** of SBPH injected with *dsLsCaM* or *dsLsenolase* compared with those injected with *dsGFP* (*P < 0.05, **P < 0.01; Student’s *t*-tests).

The conserved salivary proteins CaM, enolase, stubble-2, placental protein 11 (PP11), α-*N*-acetylgalactosaminidase (NAGA), carboxylesterase, regucalcin, and trypsin-26 were selected for RNAi application in SBPH nymphs. Phenotypic variation was observed at 24 h intervals. Morphological defect and lethality were virtually undetectable in the *dsGFP*-injected SBPH nymph control throughout the test period. The suppression of *LsCaM* or *Lsenolase* transcription was lethal to SBPH (Figure 1). Compared with the *dsGFP*-injected SBPH nymphs, the *dsLsenolase*-injected and *dsLsCaM*-injected nymphs presented with significantly lower survival rates 3 and 4 days after dsRNA injection, respectively. At 7 days post-injection, their survival rates were 22 and 62%, respectively (Figure 1A). By contrast, suppression of the other six salivary protein-encoding genes did not cause any abnormalities in the SBPH nymphs (Figures 1A,B).

The qPCR analysis confirmed that the expression levels of all eight genes were markedly lower in the RNAi-treated SBPH nymphs than in the *dsGFP*-injected nymphs (Figures 1C,D and Supplementary Figure 1). The *Lsenolase* and *LsCaM* transcript levels were ∼70% lower at 2–6 days post-injection (Figures 1C,D).

### Knocking Down *LsCaM* Impairs Small Brown Planthopper Feeding and Fecundity

**FIGURE 2:**
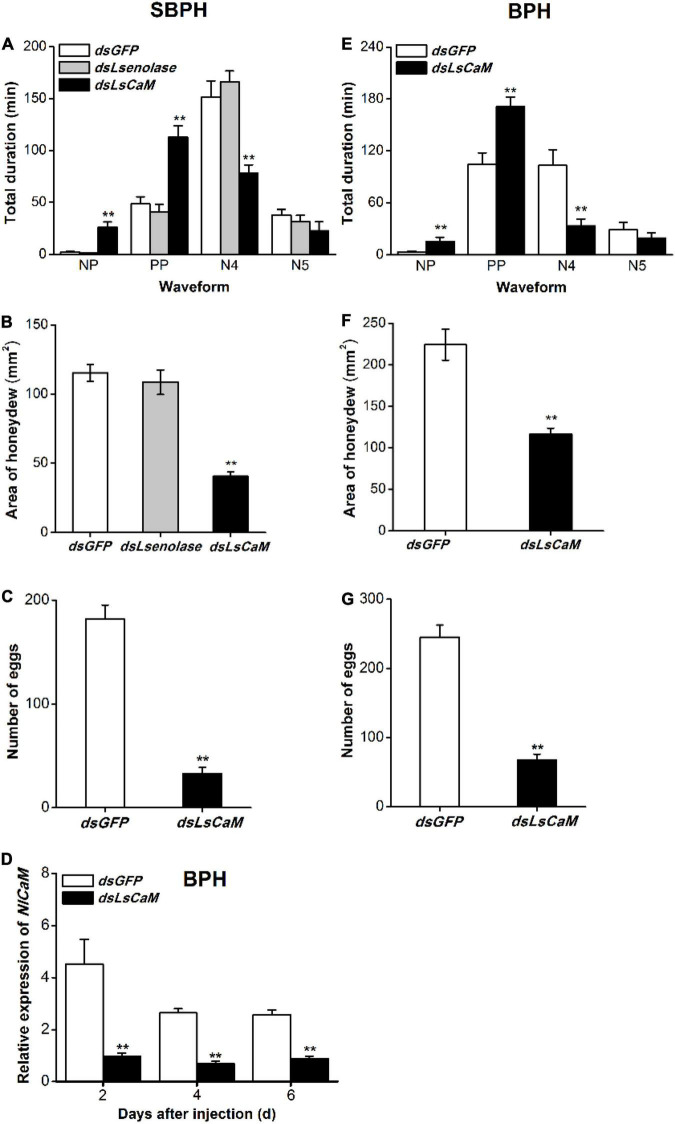
Calmodulin knockdown reduced feeding and fecundity of SBPH and BPH. **(A,E)** Mean duration + SE (*n* = 15) at different feeding phases of newly emerged SBPH **(A)** or BPH **(E)** female adults. Fifth-instar nymphs were injected with either indicated dsRNA 2 days before the EPG test. EPG waveform: NP, non-penetration; PP, pathway phase including penetration initiation, salivation and stylet movement, and extracellular activity near the phloem; N4, phloem phase; N5, xylem phase. **(B,F)** Mean area + SE (*n* = 15) of honeydew excretion on filter paper by newly emerged SBPH **(B)** or BPH **(F)** female adults receiving same foregoing treatments. Area of honeydew excretion corresponds to insect feeding intake. **(C,G)** Mean number of eggs + SE (*n* = 15) laid by a female SBPH **(C)** or BPH **(G)** adult receiving same foregoing treatments. **(D)** Mean *NlCaM* transcript levels + SE (*n* = 3) in BPH 2, 4, and 6 days after *dsLsCaM* or *dsGFP* injection. Asterisks indicate significant changes in feeding behavior **(A,E)**, honeydew excretion **(B,F)**, fecundity **(C,G)**, and gene expression **(D)** of planthoppers injected with *dsLsCaM* or *dsLsenolase* compared to those injected with *dsGFP* (**P < 0.01; Student’s *t*-tests).

To investigate whether Lsenolase and LsCaM are effectors, we used the EPG technique to study the effects of knocking down *LsCaM* and *Lsenolase* on SBPH feeding. EPG profiles the feeding behavior of piercing-sucking insects (Seo et al., 2009; Ji et al., 2017) and distinguishes the non-penetration (NP), pathway (penetration initiation, salivation, stylet movement, and extracellular activity near the phloem; PP), the phloem (N4), and the xylem (N5) phases. *LsCaM* silencing significantly prolonged NP and PP but significantly shortened N4 (Figure 2A). Silencing *LsCaM* also significantly reduced honeydew excretion (Figure 2B). Hence, food intake had decreased. By contrast, silencing *Lsenolase* affected neither the feeding behavior nor the amount of honeydew excreted (Figures 2A,B). Taken together, these results suggest that LsCaM is required for SBPH feeding, whereas enolase is not. The number of eggs laid by the *LsCaM*-silenced female was 82% lower than those laid by the *dsGFP*-injected female (Figure 2C).

### Sequence Analysis of Calmodulin From Brown Planthopper and Small Brown Planthopper

A BLAST analysis revealed that the CORF of SBPH *LsCaM* had 96% identity with the CORF of the previous reported BPH *NlCaM* (Wang et al., 2018; Supplementary Figure 2A). Nevertheless, they had identical protein sequences. Thus, we used dsRNA synthesized from the *LsCaM* CORF sequence to knock down BPH *NlCaM*. Injection with *dsLsCaM* downregulated BPH *NlCaM* by 66–79% at 2–6 days post-injection (Figure 2D). Therefore, high RNAi efficiency of BPH *NlCaM* was achieved through *dsLsCaM* injection.

### Calmodulin Enhances Brown Planthopper Performance

Consistent with the above finding on SBPH, relative to the *dsGFP* control, *dsLsCaM* injection into BPH significantly extended NP and PP but shortened N4 (Figure 2E). Silencing *NlCaM* (*dsLsCaM*) reduced by 35% the amount of honeydew excreted by BPH (Figure 2F). Moreover, the fecundity of *NlCaM*-silenced BPH female adult (*dsLsCaM*) had declined by 73% (Figure 2G). Thus, NlCaM is also implicated in BPH feeding, fecundity, and survival.

### Calmodulin Is Indispensable for Small Brown Planthopper and Brown Planthopper Survival on Rice

**FIGURE 3:**
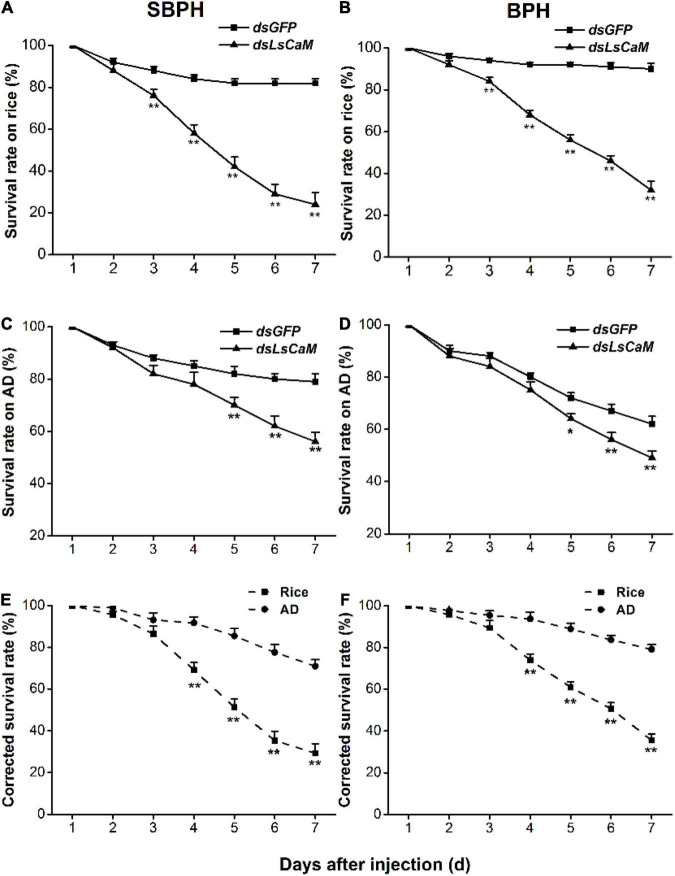
Knocking down *CaM* reduces survival rates among nymphs. **(A–D)** Mean survival rates + SE (*n* = 5) of SBPH and BPH nymphs injected with *dsLsCaM* or *dsGFP* at third-instar nymph stage, fed on rice **(A,B)** or artificial diet (AD; **C,D**). **(E,F)** Mean corrected survival rates + SE (*n* = 5) of SBPH **(E)** and BPH **(F)** nymphs with injected *dsLsCaM*, using nymphs with injected *dsGFP* as controls, feeding on rice plants or artificial diet. Asterisks indicate significant differences between treatments (*P < 0.05, **P < 0.01; Student’s *t*-tests).

To test whether CaM influenced the SBPH and BPH survival on rice and whether this influence was related to the role of CaM in the planthopper-rice interaction, we compared the survival rate of nymphs feeding on different food matrices. *CaM* silencing generally reduced the survival rate of SBPH and BPH (Figure 3), and the effect was most pronounced on rice plants: 3 days after dsRNA injection, the survival rate of SBPH and BPH nymphs on rice dropped significantly, and it was 24% (SBPH) and 32% (BPH) at 7 days (Figures 3A,B). In contrast, the survival rate of nymphs with knocked down *CaM* was higher in insects raised on artificial diet than in those raised on rice; moreover, the survival rate of nymphs with silenced *CaM* raised on artificial diet was reduced slightly compared with that of control nymphs 5 days after the start of the experiment (Figures 3C,D). Compared with that of nymphs with silenced *CaM* raised on artificial diet, the corrected survival rate of nymphs with silenced *CaM* raised on rice was significantly lower 4–7 days post-injection (Figures 3E,F). These results demonstrate that CaM contributes to the survival of SBPH and BPH nymphs raised on rice.

### Calmodulin Characterization

**FIGURE 4:**
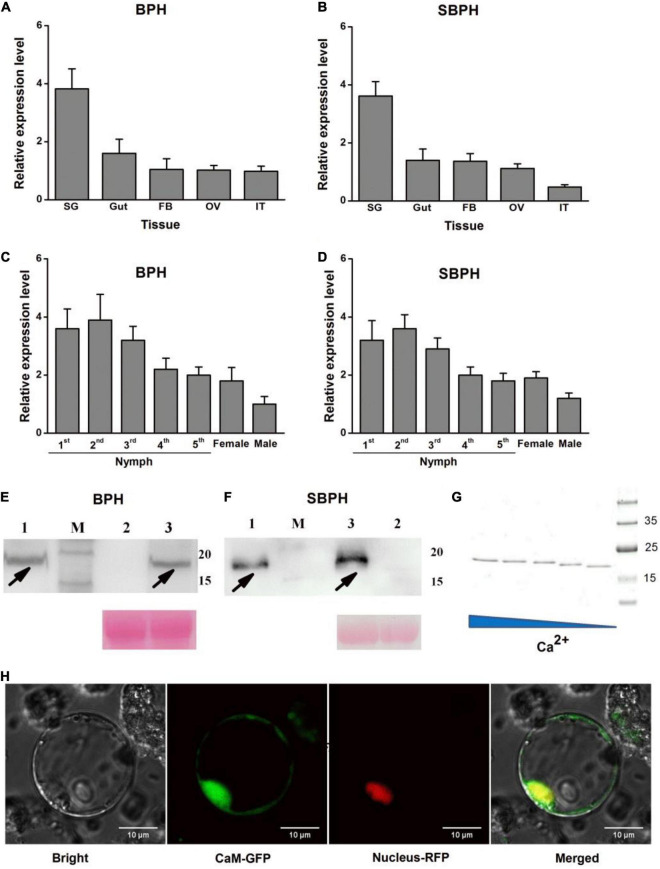
Molecular characterization of CaM. **(A–D)** Mean transcript levels + SE (*n* = 3) of *LsCaM* and *NlCaM* in BPH **(A,C)** and SBPH **(B,D)** whole bodies at various developmental stages **(A,B)** and in various tissues **(C,D)**. SG, salivary gland; OV, ovary; FB, fat body; IT, integument. **(E,F)** CaM secretion by BPH **(E)** or SBPH **(F)** into host rice. Western blot analysis of CaM. Lane 1 shows extracts of BPH **(E)** or SBPH **(F)** salivary glands. Lanes 2 and 3 represent extracts of uninfested control rice leaf sheaths (lane 2) or rice leaf sheaths infested with fifth-instar nymphs of BPH **(E)** or SBPH **(F)**. M, molecular weight marker (kDa). Equal loading of plant samples (lanes 2 and 3) was indicated by Ponceau stained-Rubisco protein (PS). Black arrowheads indicate CaM target bands. **(G)** SDS-PAGE showing Ca^2+^-dependent mobility of recombinant protein CaM. Purified CaM was incubated at 25°C for 30 min as follows: lane 1, 0.5 mM CaCl_2_; lane 2, 0.15 mM CaCl_2_; lane 3, 0.05 mM CaCl_2_; lane 4, 0.015 mM CaCl_2_; lane 5, 0.5 mM EDTA. **(H)** Transiently expressed CaM was localized to rice cell cytoplasms and nuclei. CaM-GFP fusion protein was expressed in rice protoplasts by polyethylene glycol-mediated transformation. Protein distribution was investigated by confocal laser-scanning microscopy at 16 h after transformation. Bars = 10 μm.

Spatial expression analysis revealed that BPH and SBPH *CaM* were expressed in all tissues examined and highly expressed in salivary glands (Figures 4A,B). Temporal expression pattern revealed that BPH and SBPH *CaM* were expressed at all developmental stages (Figures 4C,D).

Calmodulin protein comprises 149 amino acids, and its molecular mass is 16.81 kDa. Previous studies showed that four and five unique peptides were detected in the CaM proteins derived from BPH and SBPH watery saliva proteomes, respectively (Huang et al., 2018; Fu et al., 2021; Supplementary Figure 2B). We hypothesized that BPH and SBPH secrete CaM as they feed on rice. We performed a western blot analysis to test this hypothesis. The antibody revealed ∼16 kDa bands in plants grazed by BPH or SBPH nymphs (Figures 4E,F; lane 3). These bands were absent in uninfested control plants (Figures 4E,F; lane 2). The same bands were also detected in BPH and SBPH salivary gland extracts (Figures 4E,F; lane 1). Hence, CaM is transferred from BPH and SBPH salivary glands to the host plant during feeding.

The CaM protein had no extracellular signal peptides or transmembrane domains but contained four EF-hand Ca^2+^-binding domains (Supplementary Figure 2B). We conducted an electrophoretic mobility shift assay to determine the Ca^2+^-binding capacity of the CaM protein. Recombinant CaM was produced in an *E. coli* expression system, mixed with various CaCl_2_ concentrations, and subjected to sodium dodecyl sulfate–polyacrylamide gel electrophoresis (SDS-PAGE). CaM mobility was lower in the presence of 0.015–0.5 mM CaCl_2_ than it was in the presence of 0.5 mM EDTA. Moreover, high CaCl_2_ concentrations impeded CaM migration (Figure 4G). Similar electrophoretic mobility shift patterns were reported for other Ca^2+^-binding proteins (Hattori et al., 2012; Ye et al., 2017; Tian et al., 2021), but not for the BSA control protein. When CaM-GFP fusion protein was transiently expressed in rice protoplasts or *N. benthamiana* leaves, GFP fluorescence was detected in the cytoplasms and nuclei (Figure 4H and Supplementary Figure 3).

### Transient Calmodulin Expression *in planta* Suppresses Plant Immune Responses

**FIGURE 5:**
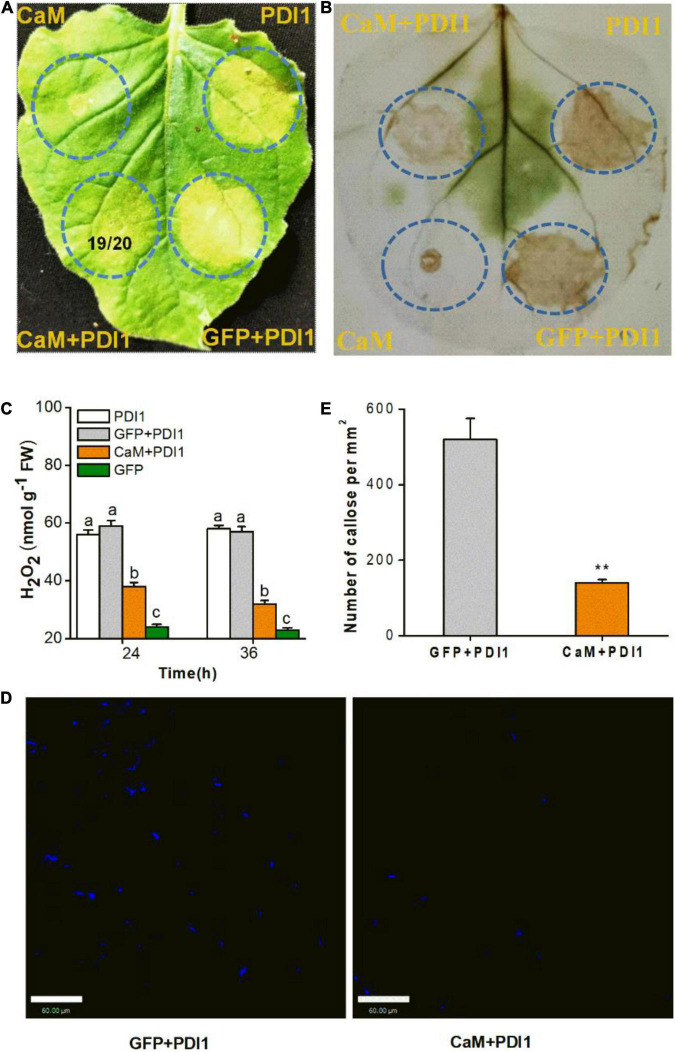
Transient CaM expression suppresses cell death, H_2_O_2_ accumulation, and callose deposition in *Nicotiana benthamiana* leaves. **(A)** LsPDI1-induced cell death was observed in all leaf areas co-expressing GFP and LsPDI1. A ratio of 19/20 in the circle of representative leaf indicates cell death reduction in 19 leaf areas co-expressing CaM and LsPDI1 relative to 20 experimental leaves. *N. benthamiana* leaves were infiltrated with *A. tumefaciens* harboring GFP or CaM at 24 h before LsPDI1 expression in the same region. Experiment was repeated on 20 leaves. PDI1 indicates the SBPH salivary elicitor LsPDI1. **(B)** Mitigation of LsPDI1-triggered H_2_O_2_ accumulation in *N. benthamiana* leaves by CaM and LsPDI1 co-expression. After DAB staining and decolorization, brown color intensity and area were commensurate with H_2_O_2_ level. LsPDI1-induced H_2_O_2_ accumulation apparent in leaf areas co-expressing GFP and LsPDI1. **(C)** Mean levels + SE (*n* = 6) of H_2_O_2_ in infiltrated *N. benthamiana* leaves receiving foregoing treatments. Experiment was repeated six times with 0.1 g infiltrated areas combined as a single sample. Different letters indicate significant difference among treatments (*P* < 0.05; one-way ANOVA followed by Duncan’s multiple range test). **(D)** Representative images of callose deposition in *N. benthamiana* leaves. Aniline blue staining of *N. benthamiana* leaves showing callose deposition (bright blue fluorescence) in areas transfected with *GFP* or *CaM-GFP* 24 h before LsPDI1 expression in the same region. Photographs were taken 72 h after infiltration. **(E)** Mean number of callose deposits + SE (*n* = 20) per mm^2^. *N. benthamiana* leaves received same foregoing treatments. Asterisks indicate significant changes (*P* < 0.01; Student’s *t*-tests). Bars = 60 μm.

Small brown planthopper salivary LsPDI1 is an elicitor that induces Ca^2+^-dependent cell death, reactive oxygen species burst, and callose deposition in plants (Fu et al., 2021). Transient CaM-GFP and LsPDI1-mCherry expression was performed in *N. benthamiana* leaves to determine whether CaM influences plant immune responses. CaM-GFP expression significantly suppressed LsPDI1-induced cell death (Figure 5A), H_2_O_2_ accumulation (Figures 5B,C), and callose deposition (Figures 5D,E), whereas only GFP expression control did not (Figure 5). Taken together, these results imply that CaM may suppress plant immune responses by binding free Ca^2+^ in the plant cell cytoplasms and nuclei.

### Calmodulin Secretion Suppresses Planthopper-Induced Callose Deposition and H_2_O_2_ Accumulation in Rice

**FIGURE 6:**
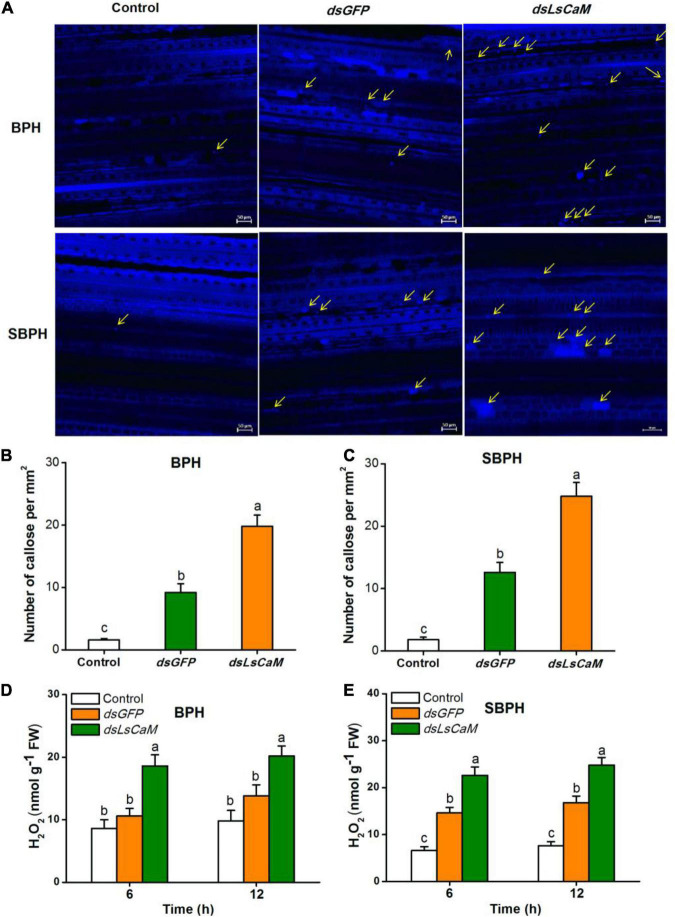
Calmodulin secreted by BPH and SBPH suppresses callose deposition and H_2_O_2_ accumulation in rice. **(A)** Representative images of callose deposition in rice leaves indicated by bright blue fluorescence. Rice leaves were infested for 12 h with 30 fifth-instar BPH or SBPH nymphs that were injected 2 days earlier with *dsLsCaM* or *dsGFP*. The uninfested rice leaves severed as controls. Yellow arrows indicate deposited callose. Bars = 50 μm. **(B,C)** Mean number of callose deposits + SE (*n* = 20) per mm^2^. Nymphs and rice leaves received same foregoing treatments. **(D,E)** Mean H_2_O_2_ levels + SE (*n* = 6) in rice plants receiving same foregoing treatments. Different lowercase letters indicate significant difference between treatments (*P* ≤ 0.05; one-way ANOVA followed by Duncan’s multiple range test).

We also measured callose deposition and H_2_O_2_ accumulation in rice plants infested with *dsGFP*- or *dsLsCaM-*injected BPH or SBPH nymphs. Callose deposition and H_2_O_2_ concentration were significantly higher in the *dsLsCaM*-BPH-infested rice plants than they were in the *dsGFP*-BPH-infested plants (Figures 6A,B,D). The same also was true for SBPH (Figures 6A,C,E). Thus, secreted CaM by BPH and SBPH may suppress insect-induced callose deposition and H_2_O_2_ accumulation.

## Discussion

Calmodulin is a member of the EF-hand family of eukaryotic calcium-binding proteins. Previous study showed that the CaM contributed to BPH survival and female adult fecundity (Wang et al., 2018). However, its salivary distribution, Ca^2+^ affinity, and effector function in planthopper-plant interactions remain unknown. Analysis of saliva proteome and western blotting demonstrated that BPH and SBPH secreted CaM into rice during feeding. Subcellular localization indicated CaM was localized to the plant cytoplasms and nuclei. CaM mobility on SDS-PAGE gel was markedly reduced by increasing the CaCl_2_ concentration due to the Ca^2+^-binding capacity *via* four EF-hand motifs. This discovery was consistent with other salivary Ca^2+^-binding proteins comprising multiple EF-hand motifs (Hattori et al., 2012; Ye et al., 2017; Tian et al., 2021). Thus, CaM may bind free Ca^2+^ after it is injected along with planthopper saliva into plant tissues, then modulate host plant defenses.

The planthoppers salivary proteomes (Huang et al., 2018; Fu et al., 2021) demonstrated that only ∼25% of salivary proteins contained an N-terminal signal peptide, which might be delivered into saliva through the eukaryotic endoplasmic reticulum Golgi pathway (Walter and Johnson, 1994). Although CaM don’t have a signal peptide, western blotting confirmed that CaM is a salivary protein. However, whether many salivary proteins like CaM without signal peptides are secreted in an unconventional way (Nickel and Rabouille, 2009) remains unknown. Transient and drastic elevation in plant cytosolic Ca^2+^ is markedly induced by diverse stimuli. In unstimulated plant cells, CaM localized to the cytoplasm and nuclei when transiently expressed. Thus, the binding of CaM and Ca^2+^ decreased free Ca^2+^ level in cytoplasm. This may interfere with the signal transduction from Ca^2+^ influx to specific defense responses. However, the function of CaM in the nuclei is unknown. The *Arabidopsis* Ca^2+^-dependent modulator of ICR1 (CMI1, Ca^2+^-binding protein) containing a EF-hand motif is localized in the plasma membrane, cytoplasm and nuclei, whereas the interaction between interactor of constitutively active ROP (ICR1) with CMI1 is Ca^2+^-dependent and make the latter be excluded from nuclei (Hazak et al., 2019). Thus, CaM binding Ca^2+^ or plant target protein also may affect its subcellular localization and function.

Elevation in cytosolic Ca^2+^ concentration is an early, vital defense-related signal. It activates downstream plant defense responses, such as H_2_O_2_ and callose biosynthesis (Hayashi et al., 1987; Zuppini et al., 2004). Callose deposition around sieve pores caused by sap-sucking insects penetrating was observed around the stylet puncture sites in rice sieve tubes, and could seal sieve pores, thereby preventing the loss of phloem sap (Hao et al., 2008). In response, insect secretes certain salivary effectors that manipulate the host plant defenses and facilitate feeding. An analogy is the mixture of salivary proteins that blood-feeding arthropods secrete to block hemostasis in their mammalian hosts. These salivary proteins inhibit platelet aggregation and blood coagulation, and ensure successful feeding. On the other hand, piercing-sucking herbivorous insects feed from sieve elements by circumventing or suppressing the host plant sieve plate sealing response. Ca^2+^-chelating agents such as EDTA prevent H_2_O_2_ and callose biosynthesis (Will et al., 2007). Analyses of saliva proteomes revealed that various Ca^2+^-binding proteins occur in several sap-sucking herbivorous insects, such as planthoppers, whiteflies, aphids, Asian citrus psyllids, and green rice leafhoppers (Huang et al., 2018; Huang H. J. et al., 2020; Fu et al., 2021). Therefore, salivary Ca^2+^-binding proteins may be ubiquitous among phloem feeders and perform a common function of suppression of callose deposition and H_2_O_2_ production, resembling that of EDTA. For instance, two salivary Ca^2+^-binding proteins, SBPH LsECP1 and BPH NlSEF1, attenuate Ca^2+^-triggered H_2_O_2_ biosynthesis by binding free Ca^2+^ in host rice (Ye et al., 2017; Tian et al., 2021). Nevertheless, their functions of inhibition of callose deposition remain unknown.

Pathogen and insect infestation can induce H_2_O_2_ accumulation in plant that is harmful for the parasites. On the other hand, H_2_O_2_ acts as a common stress signal and triggers various plant defense responses such as programmed cell death, phytohormone biosynthesis and callose deposition (Kong et al., 2013; López-Cruz et al., 2017). Callose synthesis occurs at the site of H_2_O_2_ generation (Iwano et al., 2002), and H_2_O_2_ induces callose deposition, probably by activating expression of callose synthases (Bolwell et al., 2002). Expression of CaM in tobacco leaves could significantly inhibit callose deposition and H_2_O_2_ accumulation induced by the SBPH elicitor LsPDI1. When *CaM*-silenced planthoppers fed on rice plants, they were unable to suppress the callose deposition and H_2_O_2_ accumulation they had induced in the host plant by feeding on it. Consistent with the function of CaM, SBPH LsDNase II suppresses insect-elicited callose deposition and H_2_O_2_ accumulation in rice by degrading the extracellular DNA released by damaged plant cells (Huang et al., 2019). Aphid salivary effector Mp55 also suppresses the two foregoing defense responses [18]. Thus, CaM secreted by planthoppers may rapidly bind Ca^2+^ flowing into the sieve tubes in response to stylet puncturing and prevent increases in the sieve tube Ca^2+^ concentration. This mechanism may suppress the callose deposition directly, or inhibit the H_2_O_2_ signal biosynthesis, thereby preventing the downstream activation of callose deposition. In the future, the detailed molecular mechanism by which CaM governs H_2_O_2_ biosynthesis and callose synthases needs to be elucidated.

Knocking down *CaM* decreased the survival of SBPH and BPH fed on rice significantly. By contrast, the silencing did not affect the early ability of planthoppers to feed on artificial diet without callose and H_2_O_2_-mediated plant defense (i.e., for the first 5 days). Moreover, CaM is highly expressed in salivary glands of SBPH and BPH. These results suggest that CaM acts as a novel salivary effector to suppress defense responses in rice. Interestingly, CaM was also found to be expressed in all other tissues examined, suggesting that CaM may have other biological functions in planthoppers, i.e., the CaM may play roles in BPH nymphs and ovaries development (Wang et al., 2018). In other insects, silencing *CaM* affects the responsiveness of insects to odorants (Bahk and Jones, 2016). CaM also plays an important role in vitellogenesis and in the taste response of the sugar receptor (Brown et al., 2010; Brubaker-Purkey et al., 2013). Thus, CaM is likely to be a multifunction protein, and further research will be necessary to elucidate other roles in planthoppers.

The present study demonstrated that planthoppers secreted a classical Ca^2+^-binding salivary protein into the cells of the plants upon which they were feeding. This mechanism facilitated continuous feeding by suppressing host plant defense responses. Planthoppers use effector proteins to enhance their predation on plants by suppressing the H_2_O_2_ signaling pathway and callose deposition in the host. These findings lay theoretical and empirical foundations for breeding and propagating *CaM* dsRNA transgenic rice that are resistant to predation by sap-sucking insects. They also clarify the evolutionary mechanism by which insect salivary effectors suppress host plant immunity through “group warfare.”

## Data Availability Statement

The original contributions presented in the study are included in the article/Supplementary Material, further inquiries can be directed to the corresponding authors.

## Author Contributions

RJ and JFa conceived the research. RJ, JFu, YS, LW, TT, JL, LG, ZZ, and MJ performed the experiments, analyzed the data, and wrote the manuscript. All authors contributed to the article and approved the submitted version.

## Conflict of Interest

The authors declare that the research was conducted in the absence of any commercial or financial relationships that could be construed as a potential conflict of interest.

## Publisher’s Note

All claims expressed in this article are solely those of the authors and do not necessarily represent those of their affiliated organizations, or those of the publisher, the editors and the reviewers. Any product that may be evaluated in this article, or claim that may be made by its manufacturer, is not guaranteed or endorsed by the publisher.

## Footnotes

1
http://www.cbs.dtu.dk/services/SignalP/

2
http://www.cbs.dtu.dk/services/TMHMM/

3
http://prosite.expasy.org/
