# Supplementary material for: Planthopper-Secreted Salivary Calmodulin Acts as an Effector for Defense Responses in Rice
Source: Front Plant Sci. 2022 Feb 28;13:841378. doi: 10.3389/fpls.2022.841378 (PMC8918949; doi:10.3389/fpls.2022.841378)
Supplement: Supplementary file 1 [file Data_Sheet_1.pdf]

Supplementary Materials

Table S1. Primers used in this study.

Gene	Forward (5'-3')	Reverse (5'-3')
**qPCR**		
LsCaM	GTAAACAAGACCGCACCCAG	TGTTAGCACTGTAGACGCCA
NlCaM	ATGGCACCATCACCACCAAA	ACTCGGGGAAGTCGATTGTG
Lsenolase	GGTTACAGTCAAATCGTCTCC	TCTATCAGGGCTTCATCAAGG
Lsstubble-2	CAACTTATCCGTGTCAAATGCT	TGTCACCATCACAAGAATCTACAC
LsNAGA	CTGAACTGAGTGGTGAAGAG	GCGAGGTATTTGAAGTGTGG
LsPP11	TGTAGTAAGAGTTGTCAGTGGT	GGCTGTAATTGTAGGATTCTGG
Lscarboxylesterase	GACTTGAATGTGATGGCGTG	AATGGATTGGAGTGTATGATGG
Lstrypsin-26	CTGTTCTCCATCTGTTATGCTG	CATTCTTGCCTCCACTAATCC
Lsregucalcin	TCCTACTCTTCACATCAACTTCAG	GGCAGTATTCAGTTTCTTAGTGG
ef2	GTCTCCACGGATGGGCTTT	ATCTTGAATTTCTCGGCATACATTT
**RNAi**		
dsLsCaM	TAATACGACTCACTATAGGATGGCCGACCAACTGACAGAGG	TAATACGACTCACTATAGGTCACTTGGATGTCATCATTG

dsLsenolase	TAATACGACTCACTATAGGCTAGGGTGAGGGATAGAACTG	TAATACGACTCACTATAGGAGATACTGAGGATTGAGGAGG
dsLsstubble-2	TAATACGACTCACTATAGGACAATCTCAGTATCAGAACTTGGG	TAATACGACTCACTATAGGTGAACATCCAAATCTACGGTC
dsLsNAGA	TAATACGACTCACTATAGGCCAGCCGAACCATATACCCA	TAATACGACTCACTATAGGCACTCTTCACCACTCAGTTCAG
dsLsPP11	TAATACGACTCACTATAGGGCTTTATTTGACAACTACGACC	TAATACGACTCACTATAGGGTAAACCCAATTGTGAAGTCCA
dsLscarboxylesterase	TAATACGACTCACTATAGGGCTGACCCAACTTGAATGAG	TAATACGACTCACTATAGGCATAAAGAGACACAGAGGAGAC
dsLstrypsin-26	TAATACGACTCACTATAGGAAGAAATGGCTTATCACTGGAG	TAATACGACTCACTATAGGTTTCCCATACACTAGAGGACTG
dsLsregucalcin	TAATACGACTCACTATAGGTGCTGACGTTTCTATTCCTGAC	TAATACGACTCACTATAGGTTCACCACCAAATGCCACAC
dsGFP	TAATACGACTCACTATAGGCTAGTCATCTGCACCTTCTG	TAATACGACTCACTATAGGGAGTCGTTACATATCTGCCC
Expression in planta		
LsCaM-GFP	CGGGGTCGACGGATCCATGGCCGACCAACTGACAGAG	TGCTCACCATGGATCCCTTGGATGTCATCATTGTAACG
LsPDI1-cherry	CGGGGTCGACGGATCCATGGATGAAGAATCAACCAAAAGTA	TGCTCACCATGGATCCCAACTCGTCCTTTCTCGGAAGG

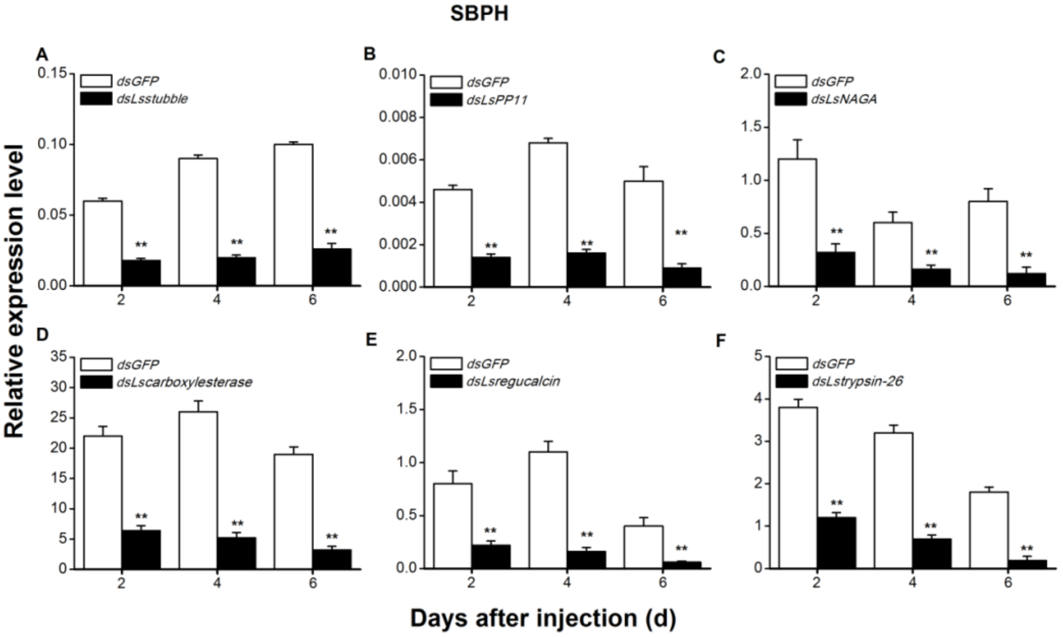

Figure S1. Mean transcript levels + SE (*n* = 6) of indicated target genes in small brown planthopper (SBPH) injected with dsRNA of *GFP* (*dsGFP*) or indicated target gene. PP11, placental protein 11; NAGA, α-*N*-acetylgalactosaminidase.

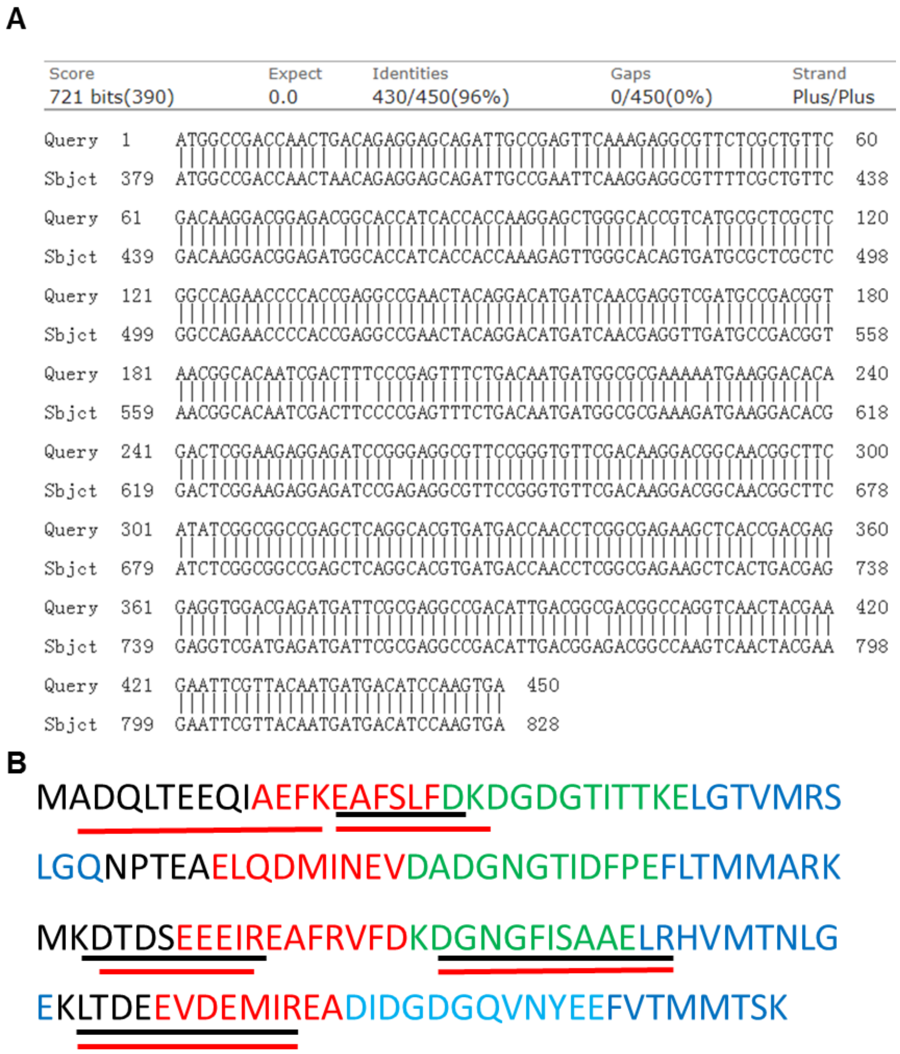

Figure S2. CaM nucleotide and amino acid sequences analyses. (A) Sequence alignment of the complete open reading frame (CORF) in *LsCaM* and *NlCaM*. CORF sequence in *LsCaM* was used in dsRNA synthesis. (B) Pivotal regions of E helices, Ca^{2+}-binding loops, and F helices in CaM represented by red, green, and blue, respectively. Underlined black and red sequences indicate peptides detected by shotgun LC-MS/MS analysis in SBPH and brown planthopper (BPH) watery saliva, respectively.

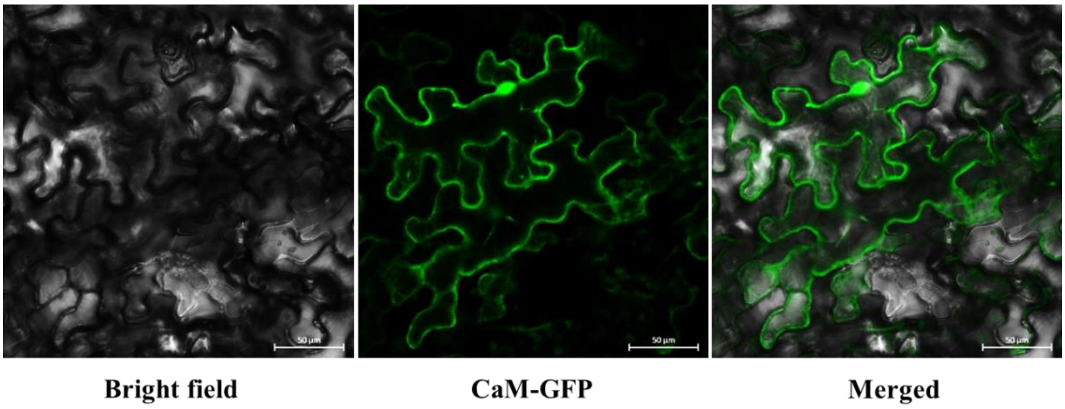

Figure S3. CaM cellular localization in *Nicotiana benthamiana* leaves. CaM-GFP expressed in *N. benthamiana* leaves via *Agrobacterium*-mediated transient transformation. Bars = 50 μm.
